# Rumen impaction in cattle associated with ingestion of the pupal cocoons of *Gonometa* spp. in Botswana

**DOI:** 10.4102/jsava.v90i0.1989

**Published:** 2019-10-10

**Authors:** Nlingisisi D. Babayani, John F. Nyange

**Affiliations:** 1 Ecosystems Dynamics, Okavango Research Institute, University of Botswana, Maun, Botswana; 2 Department of Pathology, Faculty of Veterinary Diagnosis and Research, National Veterinary Laboratory, Gaborone, Botswana

**Keywords:** rumen impaction, *Gonometa* spp., cocoons, bloat, cattle

## Abstract

Mortality in cattle associated with ingestion of cocoons (*matlhoa* in Setswana) of both *Gonometa postica* and *Gonometa rufobrunnea* is rare and has only previously been reported in South Africa, Zimbabwe and Namibia. A case history of gradual weight loss, bloat, dyschezia with dry faeces and laboured gait, resulting in sudden death after drinking water and associated with ingestion of pupal cocoons of *Gonometa* spp., was reported by keepers at Mmaditau crush in Botswana in 2013. The crush was a shared holding in a communal area with 15 registered animal keepers. The objective of this study was to profile the history, clinical signs, post-mortem findings, morbidity and mortality from the outbreak using the descriptive study method. Altogether, 81 cattle out of a total of 507 died of impaction from August to December 2013. On autopsy, a loosely connected mass of ingesta, intertwined in ropy silky strands, was observed. It was concluded that there is no readily accessible and available form of treatment at crush level, leaving only evasive husbandry practices as the feasible option. To aid evasive husbandry management practices, temporal and spatial monitoring of population dynamics of *Gonometa* spp. is recommended, particularly during a drought spell when animals are prone to develop pica, as the basis for an early warning system to farmers.

## Introduction

Rumen impaction results from accumulation of indigestible ingesta in the rumen with limited aboral movement thereof. Such material could be urban and suburban garbage like plastics (Abdullahi, Usman & Mshalia 1984; Igbokwe, Kolo & Egwu 2003; Vanitha et al. 2010) or bushveld materials like cocoons (Bafana 2009). A cocoon (pupal stage) is the sessile and over-wintering stage in the life cycle of moths and other insects. In *Gonometa* spp., the pupal stage follows from the final instar larval stage (caterpillar) about 5 weeks after the egg hatches, and in the context of Botswana’s climatic conditions it appears in September or October shortly after the first summer rains. The second generation of moths from the September and October cocoons appears in January and February and their offspring later produce cocoons at the end of March and early April. Owing to the onset of winter, cocoons produced in March and April and those that did not hatch in January and February enter diapause. This is a dormant stage that ends into the following September and October with a rise in daily minimum temperature. The predominant woody host species utilised by *Gonometa postica* and *Gonometa rufobrunnea* are acacia and mopane trees, respectively (Hartland-Rowe 1992; Veldtman 2004). Despite the fact that a pair of moths is prolific enough to produce 200 eggs per laying stage, there are biotic (density-dependent) and abiotic (density-independent) factors that influence the abundance of cocoons such that their population fluctuates rather than expands continuously (Hartland-Rowe 1992). A notable increase in cocoon densities has been documented to occur during drought periods (Edwards 1935). The influence of drought is thought to be indirect by adversely affecting population-limiting biotic factors like parasitoids, disease-causing organisms, predators and other living things that compete for food, for example, *phane* (larvae of *Gonimbrasia belina*) (Hartland-Rowe 1992). In the late 1980s, Shashe Silk (Pty) Ltd. was established to commercially produce natural silk from cocoons harvested in northeastern Botswana. Between 1986 and 1987, the company managed to procure 430 tonnes of cocoons from rural people for processing (Hartland-Rowe 1992). However, such a large-scale undertaking became commercially unviable owing to the decrease in cocoon abundance and fall in the international silk price (McGeoch 2000), so that the only current use of empty cocoons in the country is in making traditional dancing rattles on a small commercial scale at household level. Although cattle have been reported to eat cocoons elsewhere, particularly occupied ones, because of their juicy nature (Edwards 1935), this, to the authors’ knowledge, is the first report of a confirmed case of rumen impaction in cattle associated with the consumption of *Gonometa* spp. cocoons in Botswana. The aim of this study is to document the history, clinical signs, post-mortem findings, morbidity and mortality, and further discuss potential management practices that can be adopted in the context of the country’s extensive communal rearing system to avoid or limit future cases.

## Case presentation

**FIGURE 1:**
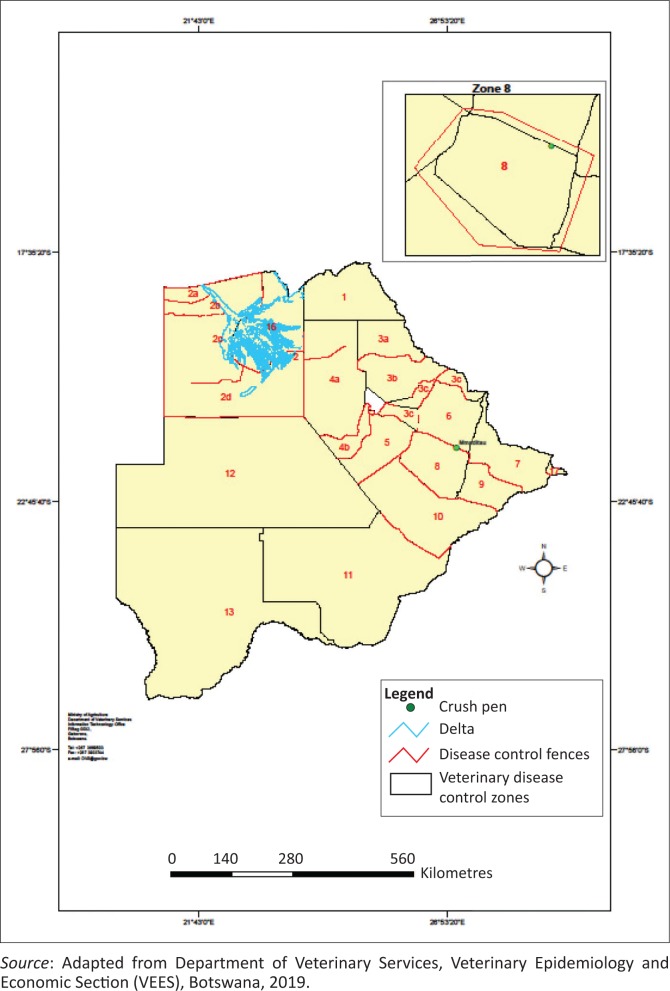
Map of Botswana showing disease control zones and location of Mmaditau crush.

Cattle were reported to be dying in increased numbers at Mmaditau crush in Zone 8 in the Central District of Botswana during the later months of the year 2013. The first death was reported to have occurred in August 2013, climaxing in mid-December 2013 and tapering to a halt in mid-February 2014. Related deaths with similar clinical signs were also reported in contiguous zones 6 and 7 (Figure 1). An epidemiological investigation was carried out in early February 2013 at Mmaditau crush to establish the probable cause of death in the cattle. The crush is located predominantly in mopane woodland with few acacia trees and 15 animal keepers were registered at that crush, owning a total of 507 Tswana and Tswana X Brahman cattle that were communally grazed, kraaled separately at night according to owner’s designated kraals and watered from a single centrally located borehole. It was reported that only cattle were affected at this crush and the affected animals initially presented with progressive weight loss, which most keepers attributed to drought and started supplementary feeding. This was followed by bloating, with foamy salivation and bruxism, ultimately leading to recumbency and death after drinking water. Upon slaughter for salvage value by the owners, a single mass of ingesta was reported to be present in the rumen, immersed in a straw-coloured fluid in all affected animals. It was alleged to cause skin irritation upon handling with bare hands once left to dry in the sun. In total, 81 mature cattle of various ages that were let out to pasture out of a total of 507 were reported to have died or to have been slaughtered for salvage value as a result of this condition at this crush, with no calves affected. Several animals with similar clinical signs were also reported to have died in adjoining zones 6 and 7.

Clinical examination was carried out on two heifers that were regarded as typical of the cases seen and reported in the area. The heifers were in poor body condition, walked with a laboured gait and abducted forelimbs. Asymmetric abdominal distension, most pronounced in the left paralumbar fossa and indicative of bloat, was evident in both heifers. Straining to defecate with intermittent release of scanty dry stools was seen in one heifer, indicating dyschezia. The skin tenting test in both showed moderate-to-severe dehydration with pale mucous membranes. On auscultation, laboured breathing with audible grunts and wheezes coupled with nasal flaring was evident. Reticuloruminal motility was subdued in both animals.

**FIGURE 2:**
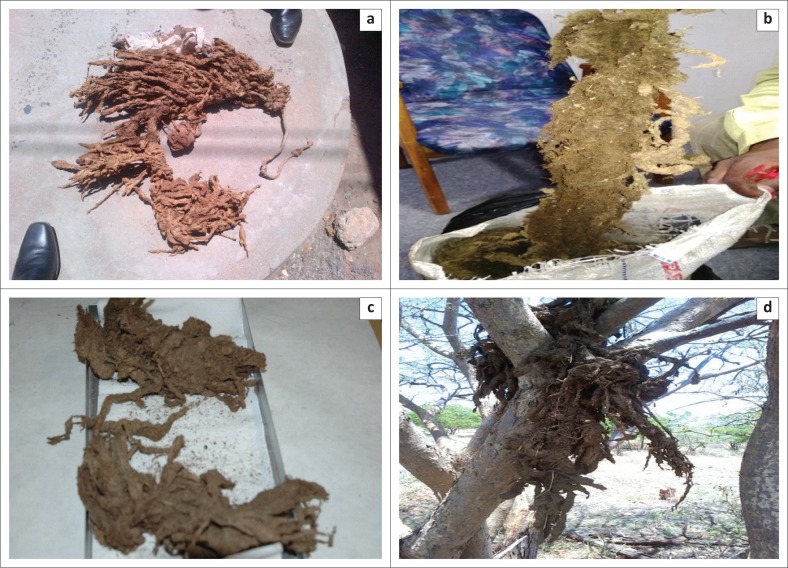
Photos of rumen impaction material at different time intervals post-sampling (a, c and d) the rumen, with (b) representing fresh impaction material as evidenced by greenish colouring.

A full autopsy was not conducted on the animals as the owners were unwilling to kill them for further investigation. However, fresh and old ingesta masses harvested from previous cases that died were availed for partial post-mortem investigation. On observation the masses consisted of ingested materials adhering to silky strands that formed irregular ropes of various dimensions (Figure 2). Some masses were still fresh, showing a typical dark green colour of leafy material, while others that were hanging from trees for some time were brown-tinged and hardened owing to desiccation from the sun. The initial speculation by farmers was that the animals ate discarded indigestible clothing materials because of prevailing drought conditions, particularly in zones 6 and 7 where a restocking exercise was underway following a foot-and-mouth disease outbreak largely with animals from distant zones that were said to be naive to edible materials in the area. Dislodging the dried ingesta from the silky strands through shaking was impossible and only beating up the ingesta mass with wooden sticks led to the disintegration of the mass. Rubbing bare skin on the dried mass caused skin irritation and submersion of the dried masses in a bucket full of water yielded brownish razor-sharp floating urticating bristles that upon decanting and drying resembled spicules found on the surface of pupal cocoons.

**FIGURE 3:**
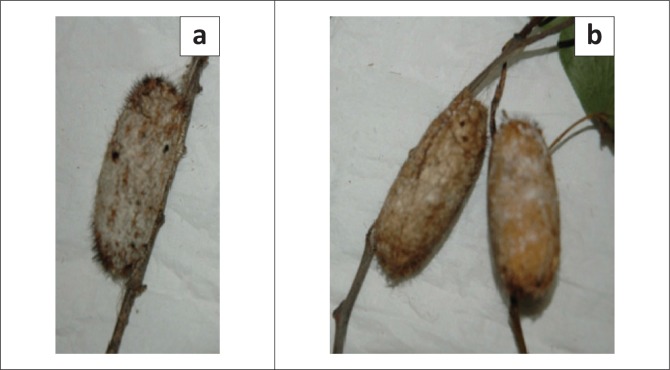
Occupied pupal cocoons of the southern African wild silkmoth species (*Gonometa* spp.) attached to twigs of acacia (a) and mopane (b) trees.

**FIGURE 4:**
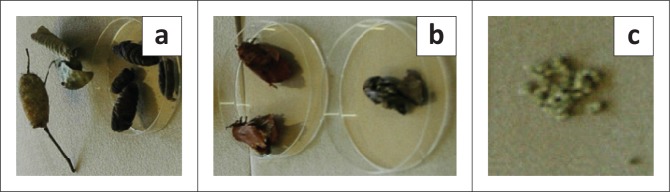
Occupied pupal cocoons (a) that were incubated leading to emergence of moths with red forewings (b) that subsequently laid white eggs with a black micropyle (c).

Environmental assessment in the vicinity of Mmaditau crush showed a high density of occupied and empty pupal cocoons of *Gonometa* spp., largely on mopane and acacia trees (Figure 3). Occupied cocoons were collected and incubated at the National Veterinary Laboratory in Gaborone and hatched to release moths with red forewings that later laid white eggs with a black micropyle (Figure 4).

## Ethical considerations

No approval was required as this case report describes observations made during a clinical case attendance by employees of the Department of Veterinary Services, who are mandated through relevant statutes to do it as part of their routine extension service to farmers in the country.

## Discussion

Mortality in cattle associated with the ingestion of cocoons of both *G. postica* and *G. rufobrunnea* has only previously been reported in South Africa (Edwards 1935; Zumpt 1971), Namibia (Veldtman 2004) and Zimbabwe (Bafana 2009). The history and clinical picture from the cases attended at Mmaditau crush is consistent with recorded findings from cases reported in South Africa and Namibia, confirming the cause of death to be rumen impaction associated with ingestion of pupal cocoons of *Gonometa* spp. Laboratory incubation of harvested cocoons confirmed the causal agent to be *G. rufobrunnea* as the emerged moths exhibited typically red forewings and laid white eggs with a distinctive black micropyle (Hartland-Rowe 1992). From previous ecological studies, the species is known to exist, and to have historically reached high population densities in the area (Hartland-Rowe 1992; Veldtman 2004).

Pathogenesis associated with ingestion of cocoons is insidious. After ingestion, cocoons disintegrate in the rumen owing to both the actions of rumen acids and the mechanical effect of reticuloruminal motility, resulting in adhering and entangling of ingesta in the loosening silk strands. With stretching of the fine silk strands from degummed cocoons, ingesta adherence progresses gradually and they clump into a large mass causing rumen impaction (Zumpt 1971). The impacted material, while still part of the mass in the rumen, has been noted by Zumpt (1971) to stretch anteriorly to lodge and block the cardia. This is the probable cause of rumen tympany (bloat) and the resultant laboured gait that was evident in all the cases as narrowing of the oesophageal lumen by impacted material would compromise eructation, leading to free-gas accumulation in the rumen (Leek 1983). Pressure on the wall of the rumen caused by foreign bodies is known to cause sloughing of the ruminal lining, leading to an inflammatory response and generalised oedema (Vanitha et al. 2010; Zumpt 1971). Pain associated with sloughing of the ruminal lining and inflammatory response could have caused the observed bruxism, leading to the drooling of foamy saliva. Furthermore, this could have caused damage to the vagus nerve endings along the rumen lining and that coupled with probable reticuloruminal smooth muscle relaxation as a result of generalised oedema, decreasing sensory input from tension receptors (Leek 1983), might have caused the noted severe reduction in reticuloruminal motility and eventual death.

The intake threshold of cocoons needed to cause clinical signs and eventual death is not known. However, industrial processing of *Gonometa* spp. cocoons has enabled an estimation of the number of cocoons needed to spin 1 kg of spun silk to be 2326–4762 (Kioko, Raina & Mueke 1999). From this, it would have been easy to estimate the quantities of cocoons that each case had consumed before it succumbed, but the difficulty encountered in trying to separate the entangled ingesta from the silk strands made it impossible to accurately measure the weight of spun silk making up the ingesta mass.

The prognosis for rumen impaction cases associated with the ingestion of pupal cocoons of *Gonometa* spp. is grave as cases end up dying, unless surgical intervention is initiated to remove the impacted ingesta mass (Edwards 1935; Zumpt 1971). However, rumenotomy is not practical as it cannot be used routinely to manage such a volume of cases in rural Botswana owing to costs associated with it, limited skilled manpower in the form of veterinarians to perform the surgery and the extensive nature of husbandry, which sometimes leads to some sick animals dying at pasture without being noticed. This therefore leaves evasive management as the only viable option that can be adopted to limit the effect at times of explosive population growth of cocoons. This might involve moving animals to cocoon-free areas for grazing, but that has the limitation of land shortage as well as drinking water availability because most animals are watered from fixed water sources, for example, boreholes, dams, rivers, and so on, that cannot be moved along with cattle. A more practical evasive approach will be to harvest cocoons at determined times of plenty as established from an objective cocoon surveillance plan. Such a move can resuscitate the once profitable wild silk industry in the country that collapsed as a result of low cocoon numbers, creating an additional source of income to farmers and employment opportunities to rural communities in the area.
